# Use of digital technologies to combat loneliness and social isolation: a cross-sectional study in Swiss outpatient care during COVID-19 pandemic

**DOI:** 10.1186/s12912-022-00946-7

**Published:** 2022-07-08

**Authors:** Sabrina Stängle, Franzisca Domeisen Benedetti, Hannele Hediger, Mathias Bonmarin, Martin Loeser, André Fringer

**Affiliations:** 1 ZHAW – Zurich University of Applied Sciences, School of Health Professions, Institute of Nursing, Katharina-Sulzer-Platz 9, 8401 Winterthur, Switzerland; 2 ZHAW – Zurich University of Applied Sciences, School of Engineering, Institute of Computational Physics, Technikumstrasse 71, 8401 Winterthur, Switzerland; 3 Department of Computer Science, Electrical Engineering and Mechatronics, ZHAW – Zurich University of Applied Sciences, School of Engineering, Technikumstrasse 9, 8401 Winterthur, Switzerland

**Keywords:** Ambulatory care, Survey, Digital technologies, COVID-19 pandemic, Switzerland

## Abstract

**Background:**

There is limited data on the use of digital technologies in outpatient care in Switzerland. Our objectives were therefore to determine which digital technologies are used and whether they had an impact on loneliness and social isolation in the wake of the COVID-19 pandemic.

**Methods:**

A cross-sectional survey design was used with a convenience sample of 1272 outpatient care providers in Switzerland. The questionnaire used is based on an unsystematic literature review and a previous qualitative study with six outpatient caregivers and two caring relatives, based on which the 30 items for this questionnaire were developed. Data were analyzed descriptively, and group comparisons were made using the Kruskal Wallis test. Changes over time were measured using Friedman test with Bonferroni post hoc tests and Wilcoxon test for paired samples.

**Results:**

The impact of the COVID-19 pandemic was evident both on the part of the health care system, e.g., inadequate protective equipment; on the part of health care providers, e.g., increasing fatigue in keeping abreast of the virus as the pandemic progressed; and on the part of clients, who reduced services of care, e.g., out of fear of infection. According to the assessment of the outpatient caregivers, loneliness and social isolation of the clients was high in spring 2020 and increased strongly in the following winter. Alternative solutions, such as digital technologies, were hardly used or not used at all by the clients.

**Conclusions:**

The results suggest that the pandemic is dramatically impacting clients. This highlights the urgent need to invest in the development of appropriate digital technologies reducing the impact of social isolation and loneliness and the associated long-term costs to the healthcare system.

**Supplementary Information:**

The online version contains supplementary material available at 10.1186/s12912-022-00946-7.

## Background

National and international studies have demonstrated that social isolation and loneliness are common among older people in need of care [1–3]. Loneliness and social isolation not merely affect older persons in need of care and their family caregivers, but also challenges health care professionals (HCP), especially nursing professionals in the home care setting. From outpatient caregivers’ point of view, loneliness can be the cause of a domestic crisis that needs to be dealt with [4].

Loneliness and social isolation are related concepts and not current only in the COVID-19 pandemic. Social isolation refers to the separation of individuals from important caregivers, groups, activities and social situations that subsequently interfere with a person’s social processes [5]. Antecedents for social isolation are individual perception and the situational dimension. The consequences of isolation are described as anxiety, depression, mood disorders, anger, loneliness, and health impairment. Therefore, social isolation may be individually perceived, and loneliness may occur as the subjective feeling of being alone [5, 6].

Social bonds, networks, integration, and primary group relations are crucial concepts to social isolation and support. These in turn are important principles for and affect both, physical and mental health [3, 7–9]. They are associated with increased mortality [10, 11] and suicide risk [12, 13] and dementia (e.g. Alzheimer’s Disease) [14]. Social isolation and loneliness as a consequence, significantly lead to increased healthcare expenditure [8, 15–18]. As a result, social isolation and loneliness are gradually being recognized as significant public health problems [19], and led, for example, to the establishment of the Ministry of Loneliness in the United Kingdom. The issue occurs in all age groups and social classes but increases in old age (> 75 years) [20]. Social distancing during the COVID-19 pandemic to protect vulnerable groups increases the risk of social isolation and feelings of loneliness and their consequences not only for the elderly people directly affected, but also for their families and health care providers. Social isolation and loneliness are impacted as follows:

The longer the contact restrictions last, the more often it is accompanied by psychological stress, avoidance strategies and anger [21–24]. Confinement, loss of usual routine, the change of habits, and reduced social and physical contact with others were frequently shown to cause boredom, frustration, and a sense of isolation from the rest of the world, which is distressing to participants [21, 22, 25, 26].

Elderly persons in need of care are especially vulnerable and therefore experience special protection, which is reflected in the fact that contacts with the outside world are greatly reduced [27]. For fear of becoming infected [21, 28], visits by outpatient care providers are canceled.

As a consequence, the pressure and responsibility on the caring relatives is greatly increased [29], also, under certain circumstances, activities are taken over that the relatives have not learned. This can lead to the person in need of care, e.g. a wound, not being treated professionally, leading to a hospital stay being necessary [29]. Caring relatives themselves belong to a vulnerable group of people who suffer to a high degree from loneliness and social isolation [30, 31] and can be attributed to a lack of recognition of their services and insufficient support from professionals [31, 32].

It becomes clear that continuous care for the persons in need of care and their relatives is necessary, especially in times of greatest uncertainty by outpatient care nurses, in order to recognize or prevent domestic crises [33]. However, outpatient care service providers are themselves affected by the impact of the crisis. They suffer from staff shortages as nurses must stay at home to provide care for their children. Nurses are overworked because work schedules and rest breaks may be suspended during the pandemic. As a result, people in need of care have their care reduced or cancelled, so that the remaining work must be absorbed by caring relatives [29].

While this spiral of interdependent factors cannot be easily broken, new strategies are needed to ensure the security of care for individuals in need of care and their families. New solutions for professional home care are needed to monitor the health status of their clients, and to maintain communication to them and their relatives.

The use of digital technologies seems suitable for times of social distancing in order to ensure the security and quality of care [34]. It needs to be clarified if other modern technology is used in home care to support the persons in need of care in addition to the classic aids (e.g., emergency call bracelet) [35]. So far, little is known about if and which digital technologies are used by outpatient care service providers in Switzerland and how digital technologies are accepted by the persons concerned and what benefits these measures achieve.

## Methods

### Aims of the study

Loneliness and social isolation and their consequences also challenge outpatient caregivers (professional care provided by home care services). The objectives were to find out from outpatient caregivers’ perspective (1) how they experienced the situation during contact restriction for their clients; (2) what challenges they faced and their consequences for their clients; and (3) what new or additional digital technologies were used, whether for communication and monitoring, e.g., to provide a sense of safety.

### Study design and setting

This is a cross-sectional study that also includes retrospective questions covering the COVID-19 pandemic period. Outpatient caregivers from all over Switzerland were included in this study. A special feature in Switzerland is the diversity of languages, including German (dominant language in the major regions: Northwestern Switzerland, Zurich, Eastern Switzerland, and Central Switzerland), French (dominated language in the major regions: Lake Geneva region and Espace Mittelland) and Italian (dominated language in the major region: Ticino) (Fig. 2). These seven major regions consist of one or more cantons with an average population density of 1,041,144 [37].

Qualitative interviews with the target group provided the basis for the development of the questionnaire by exploring their experiences. To address the specifics of the challenging COVID-19 pandemic time, the “Health Systems Resilience” of Sagan, Thomas [38] was used as a theoretical framework. It is divided into four levels.

- Level 1 - Preparedness: Addresses the extent to which the health system is prepared to deal with the unexpected situation, in this case the COVID-19 pandemic outbreak.
- Level 2 - Shock onset and alert: Here the focus is on the timely recognition of the state of crisis.
- Level 3 – Shock impact and management: Focuses on handling and dealing with the crisis.
- Level 4 – Recovery and learning: Here we reflect on the extent to which the health care system has made or still needs to adjust and what lessons can be learned for further crises.

### Population and sample

**Fig. 1:**
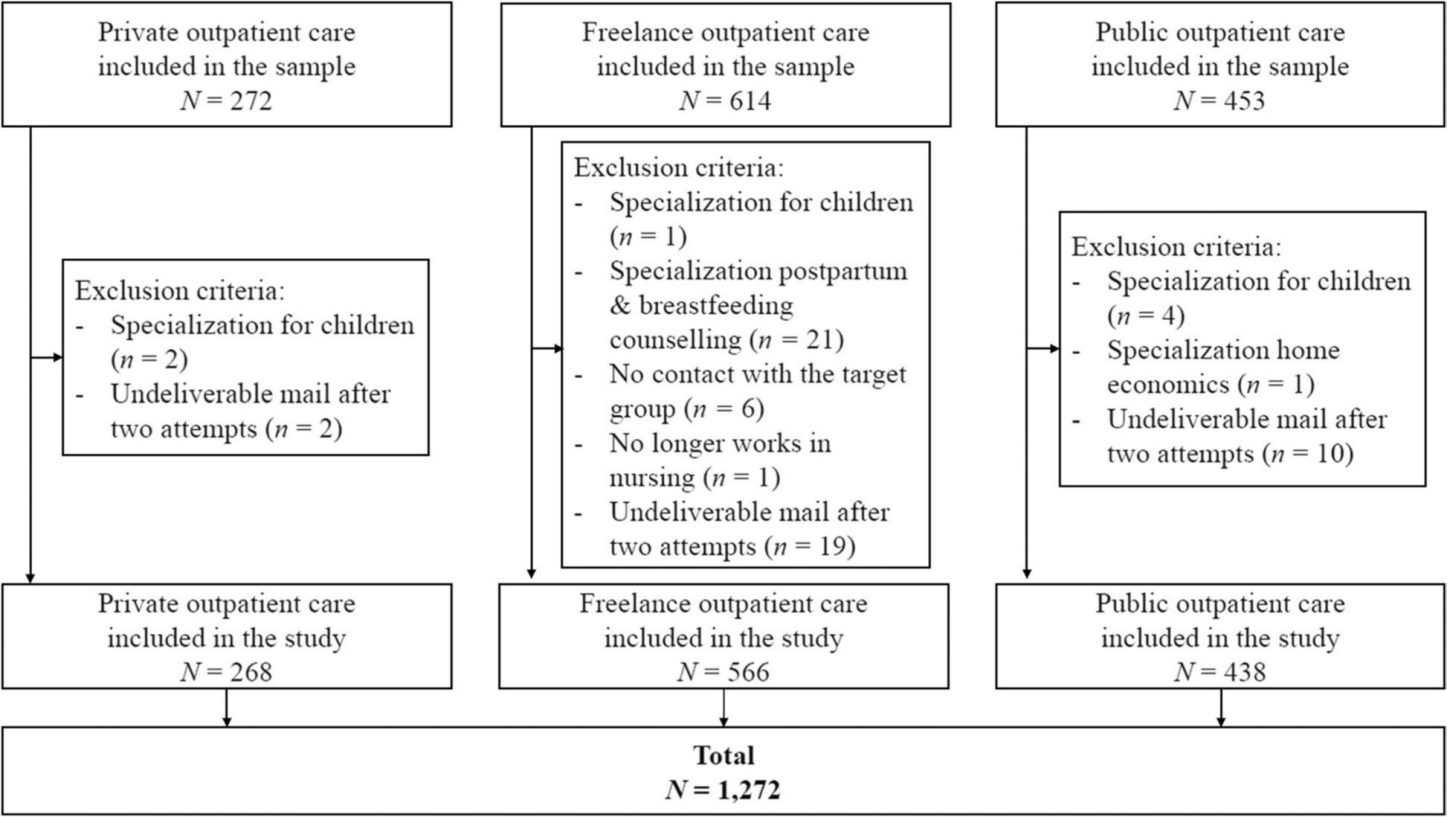
Study flow chart

Outpatient caregivers were accessed through membership lists of professional organizations available on the Internet, including private (*N* = 272; https://spitexprivee.swiss/de/), freelance (*N* = 614; https://www.curacasa.ch), and public outpatient care providers (*N* = 453; https://www.curaviva.ch). With this strategy, slightly more than half (57.2%) of all outpatient care providers in Switzerland could be reached to be invited in the study [39], of which a small proportion were excluded, e.g., due to specialization in children (Fig. 1). We focused on the population goal of one person per private and public outpatient care provider, representing the institution, plus each freelance caregiver responding, and expected a response rate of at least 20%, comparable to national studies worldwide [40]. Recruitment took place between January and April 2021 and included two reminders every 21 days for non-response. The participants were invited to the survey via e-mail using the survey software REDcap 10.9.4 (2021 Vanderbilt University). After informed consent, the participants were forwarded to the survey. All questions could be changed or completely reset by a back button.

### Questionnaire and variables

For the development of the questionnaire, an unsystematic literature review on the use and benefits of digital technologies in outpatient care was conducted in April 2020. Based on the findings from the literature, two interview guides for qualitative interviews were developed. One for the target group of outpatient care (*n* = 6) and one for clients (*n* = 2). Based on the findings of the qualitative interviews (*n* = 6 with outpatient caregivers; *n* = 2 with clients/ family caregiver) in combination with the theoretical framework of “health system resilience” [38], a questionnaire for outpatient caregivers to gather their perspective with a total of 30 items was developed covering following dimensions:

- Changes in contact behavior with clients
- Retrospective assessment of loneliness and social isolation of clients
- Appropriate technologies in contact with clients
- Pandemic precautions in the Swiss healthcare system
- Personal impact of the COVID-19 pandemic

A cross-sectional survey for clients/ family caregivers has been conducted in spring 2021 and an additional paper is in preparation.

Due to the theoretical framework, specific care was taken to ensure that certain items were surveyed multiple times with the difference in measurement times, including at the beginning of the pandemic (in spring 2020), during the pandemic (in summer 2020), and finally the current situation (in winter 2020). Response options included 5-point Likert scales (1 = disagree, 2 = tend to disagree, 3 = neutral, 4 = tend to agree, 5 = agree), free response fields, and multiple responses [41–43]. At the end of the survey, five items were asked about job-related and personal items. The questionnaire was checked and adapted by three ambulatory care nurses for comprehensibility, manageability, and completeness [42]. The questionnaire, which was developed in German, was then translated into French and Italian by two independent professional translation agencies [44] and piloted by one outpatient nurse for each language.

### Statistical analysis

Descriptive analysis was conducted using SPSS (IBM, Armonk, NY, USA; Version 27). Appropriate statistical methods such as the mean, standard deviation, median, mode and frequencies and proportions were used. Furthermore, the Kruskal Wallis test was used to check whether there were differences in response behavior between the occupational groups (public, private, or freelance outpatient care) or between the seven major regions of Switzerland. Differences in response behavior between women and men were identified using the Mann-Whitney U test, and differences between the measurement times of spring, summer and winter were analyzed with the Friedman test for paired samples and with post hoc tests. Differences between the measurement times of spring and winter were analyzed with the Wilcoxon test for paired samples. Effect sizes for all tests were defined according to Cohen (1988). The significance level was set at α = 0.05. Missing values were not included in the analyses.

## Results

### Description of participants

**Fig. 2:**
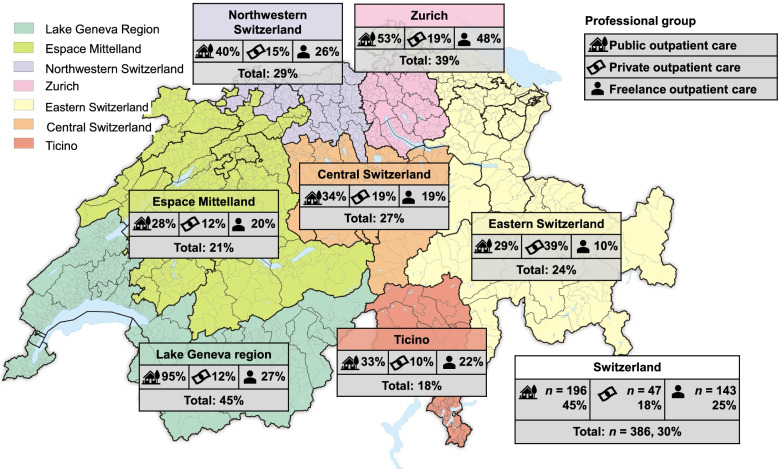
Response rate within the seven regions and Switzerland. Legend: Some participants (*n* = 14 total; *n* = 6 public outpatient care, *n* = 2 private outpatient care, and *n* = 6 freelance outpatient care) did not provide information on the region. Source: Map of Tschubby [36] with information on outpatient care provided by the authors

Of all 1272 participants invited a total of 386 participants throughout Switzerland answered the questionnaire between mid-January and the beginning of April 2021 (response rate = 30%). As shown in Fig. 2, the response rate of the seven major regions ranged between 18 and 45%. Participants’ demographics are shown in Table 1.

**Table 1:** Participants’ characteristics

	Mean (SD) Range	***n*** (%)
Total participants		386 (100%)
Age (years) (missing *n =* 17)	48.4 (10.4) 20–71	
Sex (missing *n =* 14)		
- Female		311 (83.6%)
- Male		60 (16.1%)
- Diverse		1 (0.3%)
Function (missing *n* = 13)		
- Nurse		241 (64.6%)
- Institute director		79 (21.2%)
- Team leader		53 (14.2%)

### Pandemic precautions in the Swiss healthcare system

To begin, we wanted to know from participants whether the health care system was prepared for the COVID-19 pandemic. While in the spring of 2020, about half (agreement: 44.7%, *n* = 169) of the participants had a pandemic plan that could be implemented, in the winter of 2020, three quarters (agreement: 77.3%, *n* = 290) had access to one (see Table 2). At both time points, agreement is significantly higher in public outpatient care compared to freelance caregivers (*χ*^2^[2]_(spring 2020)_ = 11.685, *p* = .003, Cohen’s *f* = 0.2; (*χ*^2^[2]_(winter 2020)_ = 17.209, *p* < .001, Cohen’s *f* = 0.24) (see Supplementary file 1). Regarding the availability of protective clothing, it appears in spring there was insufficient availability (agreement: 13.7%, *n* = 52), in summer there were more protective clothing available (agreement 76.1%, *n* = 289) and in winter there was sufficient availability (agreement: 86.0%, *n* = 327). The Friedman test showed that the central tendencies of the protective clothing differed significantly at the measurement points spring, summer and winter from each other (𝝌2 (2, *N* = 378) = 538.8, *p* < .001). The effect size could be classified as very large with a Cohen’s *f* = 1.5 (Cohen, 1988). Bonferroni’s post hoc tests showed that significance was due to differences between all measurement time points (*p* < .001). Through the possibility of free text answers, we received the information that there was an incongruence between the Swiss-wide and the regional (canton and large region) regulations and that this led to uncertainty on the part of the professionals and the clients. The freelance participants criticized above all the high costs that had to be invested in protective clothing in spring and summer, which were only reduced again in winter.

**Table 2:** Protective clothing and pandemic plan

Pandemic plan and protective clothing	Strongly disagree	Disagree somewhat	Neutral	Agree somewhat	Strongly agree
In the spring of 2020, there was a pandemic plan on what I should do in the event of a pandemic outbreak. *n* = 378	18.5%	23.0%	13.8%	26.2%	18.5%
The pandemic plan could be implemented immediately in the spring of 2020. *n* = 377	17.2%	22.8%	17.0%	27.3%	15.6%
Now in the winter of 2020, there is a pandemic plan for what I should do if the number of COVID-19 infected people increases. *n* = 375	4.0%	6.9%	11.7%	38.4%	38.9%
The pandemic plan could be implemented immediately in the winter of 2020. *n* = 375	4.3%	8.5%	13.9%	35.7%	37.6%
In the spring 2020, at the beginning of the pandemic, sufficient protective clothing (e.g., mouth guards, goggles) was available. *n* = 381	52.5%	29.7%	4.2%	6.6%	7.1%
With time, over the summer 2020 was enough protective clothing. *n* = 380	5.0%	10.8%	8.2%	45.0%	31.1%
Now in the winter of 2020, there is enough protective clothing. *n* = 380	2.9%	5.3%	5.8%	27.1%	58.9%

### Personal impact of the pandemic on the participants

In spring 2020, 43.1% of participants were afraid of getting infected (Table 3). In winter, the fear of becoming infected had decreased significantly (agreement: 31%) (Z (376) = −4.846, *p* < .001, Cohen’s d_z_ = 0.32) (Table 4). Dealing with hygiene measures to avoid infection is described as familiar by more than half in spring (agreement: 50.3%) which statistically significantly increased in winter (agreement: 70.1%) (Z (378) = − 8.683; *p* < .001, Cohen’s d_z_ = 0.5). Willingness to keep up to date on the virus was significantly higher in the spring (agreement: 89%) than in the winter (agreement: 78%) (*Z* (376) = − 6.264*, p <* .001, Cohen’s d_z_ *=* 0.2). In the free response field, participants expressed that they developed health problems because of the pandemic. Many answered they had been infected themselves through work, while others were plagued by fatigue and exhaustion due to the high workload. In their private lives, the participants also reduced their social contacts to a minimum, partly so as not to endanger those around them, but also so as not to expose their clients to additional danger.

**Table 3:** Personal impact

Personal impact	Strongly disagree	Disagree somewhat	Neutral	Agree somewhat	Strongly agree
In the spring of 2020, I was afraid of getting infected. *n* = 379	23.5%	20.6%	12.9%	23.0%	20.1%
Now in the winter of 2020, I’m afraid of getting infected. *n* = 377	29.4%	22.5%	17.0%	18.0%	13.0%
By spring 2020, I felt confident in using hygiene protection concepts to prevent infection or transmission of the virus. *n* = 380	14.5%	23.4%	11.8%	30.3%	20.0%
Now in the winter of 2020, I feel confident in using hygiene protection concepts to prevent infection or transmission of the virus. *n* = 378	5.6%	9.3%	15.1%	38.9%	31.2%
In the spring of 2020, I kept my knowledge of the virus up to date. *n* = 379	4.0%	1.3%	5.8%	34.6%	54.4%
Now in the winter of 2020, I always keep my knowledge about the virus up to date. *n* = 377	5.3%	6.9%	9.8%	32.6%	45.4%

**Table 4:** Changes in personal impact between spring and winter 2020

Personal impact^a^	Spring 2020, Mean (*SD*); *Mdn*	Winter 2020, Mean (*SD*); *Mdn*	Test statistics^b^ *p*-value Effect size^c^
I was (I’m) afraid of getting infected. *n* = 376	3.0 (1.5); 3	2.6 (1.4); 2	*Z* = − 4.846; ***p*** **< .001** Cohen’s d_z_ = 0.32
I felt (feel) confident in using hygiene protection concepts to prevent infection or transmission of the virus, *n* = 378	3.2 (1.4); 4	3.8 (1.1); 4	*Z* = −8.683 ***p*** **< .001** Cohen’s d_z_ = 0.49
I always kept (keep) my knowledge about the virus up to date. *n* = 376	4.3 (1.0); 5	4.1 (1.1); 4	*Z* = −6.264 ***p*** **< .001** Cohen’s d_z_ = 0.24

^a^Scale: 1 = disagree to 5 = agree, For spring, the question was asked in past tense and in winter in present tense.,^b^Wilcoxon-test for paired samples; ^c^Cohen’s d_z_: 0.2 a ‘small’, 0.5 a ‘medium’ and 0.8 a ‘large’ effect size; *SD*=Standard deviation; *Mdn* = Median

### Changes in contact behavior with clients during the pandemic

Forty-seven participants (12.2%) stated that their contact behavior with their clients had not changed during the pandemic. The remainder (*n* = 339) identified changes on the part of the clients as well as on the part of the outpatient care itself, whereby it was possible to indicate several answers.

- *Clients reduced services:* Of the outpatient care services 219 (64.6%) clients canceled and 200 (59%) clients reduced their services. Of all outpatient care services, 264 participants reported clients’ reasons for reducing and/or cancelling services. The reasons for this were particularly fear of infection (*n* = 245, 92.8%), or because relatives took over services (*n* = 125, 47.3%), because they worked in the home office, or for other reasons (*n* = 25, 9.5%), including clients in quarantine, client insecurities, or due to financial constraints.
- *Clients increased services:* In contrast, in 46.6% (*n* = 158) of the outpatient care services, clients requested an increased need for services. Of all outpatient care services,154 participants were able to indicate the clients’ reasons for an increase in services. The reasons for this were the loss of the social network (*n* = 101, 65.6%), because relatives were afraid of infecting the mostly elderly clients or because they themselves could not implement their visits due to workload, quarantine, or other reasons. Other reasons described by more than half (*n* = 84, 54.5%) of the participants included increased illness activity, especially among clients with mental illness; the loss of a social environment meant that logistical needs, i.e., the procurement of food or the delivery of meals, had to be taken on; and, in addition, clients demanded more services because other service providers, especially in the inpatient sector, limited or discontinued their services altogether.
- *Outpatient care reduced services:* Of all outpatient care services 20.9% (*n* = 71) canceled and 32.4% (*n* = 110) reduced services provided to their clients. Of all outpatient care services, 130 reported reasons for reducing and/or cancelling services. The reasons for this were setting priorities to minimize the risk of infection (*n* = 74, 56.9%). One third (*n* = 41, 31.5%) had to deal with staff shortages and the workload was too high (*n* = 26, 20%) to provide all services equally. Only in a few cases (*n* = 11, 8.5%) did services have to be discontinued because clients did not adhere to hygiene guidelines.
- *Outpatient care increased services:* Of all ambulatory care services, half (*n* = 177, 52.2%) reported offering more services. Of all participants, 174 gave the clients’ reasons for increasing services. This was due to early discharges from inpatient facilities (*n* = 102, 58.6%), lack of alternative service providers who temporarily did not offer their services (*n* = 78, 44.8%), an increased nursing workload of COVID-19 infected individuals (*n* = 76, 43.7%), or due to the underlying disease of the clients (*n* = 63, 36.2%). Some ambulatory care services had to compensate facilities that were overloaded (*n* = 48, 27.6%).

From the free-text responses of the participants, it appears that the changes listed here were mostly not changed over a long period of time but should rather be considered temporary.

### Loneliness and social isolation of clients

Participants estimated the loneliness and isolation of clients during three seasons: Spring, Summer and Winter 2020 on a scale of 1=“no agreement to be lonely/isolated” to 5=“agreement to be lonely/isolated”. While the participants rated the loneliness and social isolation of the clients rather high for spring and winter 2020 (agreement: 86 and 85%), they rated both clearly lower for summer (agreement: 49 and 50%) (Table 5).

**Table 5:** Loneliness and social isolation

Loneliness and social isolation	Strongly disagree	Disagree somewhat	Neutral	Agree somewhat	Strongly agree
Clients felt lonely in spring 2020, *n* = 375	2.4%	7.5%	6.9%	36.8%	46.4%
Clients felt lonely in summer 2020, *n* = 374	6.7%	21.4%	23.0%	34.5%	14.4%
Clients felt lonely in winter 2020, *n* = 370	3.0%	3.5%	7.6%	39.7%	46.2%
Clients were socially isolated in spring 2020, *n* = 374	2.9%	5.3%	5.6%	35.3%	50.8%
Clients were socially isolated in summer 2020, *n* = 375	6.1%	22.9%	21.1%	34.9%	14.9%
Clients were socially isolated in winter 2020, *n* = 375	2.7%	5.3%	7.5%	36.0%	48.5%

The Friedman test showed that the central tendencies of the loneliness differed significantly at the measurement points spring (*M* = 4.2, *SD* = 1, *Mdn* = 4), summer (*M* = 3.3, *SD* = 1.2, *Mdn* = 3) and winter (*M* = 4.2, *SD* = 0.9, *Mdn* = 4) from each other (𝝌2 (2, *N* = 333) = 242.2, *p* < .001) (Table 6). The effect size could be classified as large with a Cohen’s *f* = 0.7 (Cohen, 1988). Bonferroni’s post hoc tests showed that significance was due to differences between the measurement time points “spring” and “summer” (*p* < .001) and between “summer” and “winter” (*p* < .001), while there was no significant difference between spring and winter.

**Table 6:** Changes in Loneliness and social isolation between spring, summer, and winter 2020

Loneliness and social isolation^a^	Spring 2020, Mean (*SD*); *Mdn*	Summer 2020, Mean (*SD*); *Mdn*	Winter 2020, Mean (*SD*); *Mdn*	Test statistics^b^; *p*-value; Effect size^c^
Clients felt lonely in … *n* = 333	4.2 (1); 4	3.3 (1.2); 3	4.2 (0.9); 4	𝝌2 (2) = 242.2 ***p*** **< .001** Cohen’s *f* = 0.7
Clients were socially isolated in … *n* = 372	4.3 (1.0); 5	3.3 (1.2); 3	4.2 (1.0); 4	*𝝌2 (2) = 289.3* ***p < .001*** Cohen’s *f* = 0.7

^a^Scale: 1 = disagree to 5 = agree; ^b^Friedman Test; ^c^Cohen’s *f*: 0.1 a ‘small’, 0.25 a ‘medium’ and 0.4 a ‘large’ effect size; *SD*=Standard deviation; *Mdn* = Median

The Friedman test also showed similar results for isolation as for loneliness. The central tendencies of the assessed isolation differed at the measurement points spring (*M* = 4.3, *SD* = 1, *Mdn* = 5), summer (*M* = 3.3, *SD* = 1.2, *Mdn* = 3) and winter (*M* = 4.2, *SD* = 1, *Mdn* = 4) significantly from each other (𝝌2 (2, *N* = 372) = 289.3, *p* < .001) (Table 6). The effect size Cohen’s f was 0.7 and could be classified as large (Cohen, 1988). The post hoc tests according to Bonferroni also showed here that the significance was due to the differences between the measurement times “spring” and “summer” (*p* < .001) and between “summer” and “winter” (*p* < .001).

The free response fields confirmed that clients had received fewer visits from their relatives, especially in spring and winter, and that leisure activities were limited or not offered at all during this time. In summer, this could be compensated by outdoor activities. The effects affected both young and old clients and had a negative impact on their physical and psychological constitution. Deficits in coordination and mobility were described, as well as an increase in anxiety and depression.

### Appropriate technologies in contact with clients

**Fig. 3:**
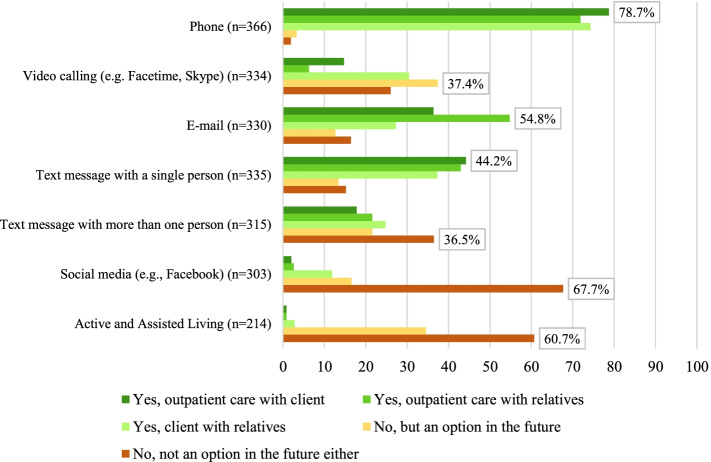
Digital technologies used in outpatient care

The following digital technologies were used in the context of outpatient care (see Fig. 3):

- In the interaction between outpatient care and clients, telephone calls (78.7%) or communication via text message (with a person) (44.2%) are usually used.
- In the interaction between the outpatient care and the relatives, there is also usually a telephone call (71.9%) or communication via e-mail (54.8%).
- Digital communication between clients and relatives usually takes place by telephone (74.3%), video calling (30.5%) or text message (with one person) (37.3%).

Participants anticipate that the use of video calling will increase in the future (37.4%). The use of social media (67.7%) and Active and Assisted Living (60.7%) is also not considered as an option for more frequent use by most participants in the future.

Two hundred and seventy-nine participants indicated barriers to the use of technology by their clients: 83.5% indicated that technologies were cognitively not understood, 57.0% stated poor usability (e.g., due to hand tremors), 55.9% no device available, 44.4% say that clients see no need, 21.1% say that purchase is too expensive, 17.9% unstable/missing internet connection and 6.8% said that data security is a barrier.

## Discussion

In this large-scale study, all questions could successfully be answered.

The objectives of this work were to learn about the experiences and challenges in ambulatory care during the COVID-19 pandemic and whether digital technologies were used during this time to overcome the situation. This study showed that from the perspective of outpatient caregivers in Switzerland social isolation and feelings of loneliness is an actual and relevant topic in COVID-19 pandemic.

International studies have shown that health systems have reached their limits in terms of pandemic preparedness [45, 46]. It also seems to be the case in Switzerland. This study indicates that in the view of participants, in particular for freelance outpatient care nurses, it led to bottlenecks in the supply of protective equipment, and they felt abandoned because they could not perform their work under the prescribed protective measures, but also received no support from the state to obtain protective equipment. International comparisons have demonstrated that there has been insufficient integration between public and private service providers, resulting in poorer care [47].

Regarding the personal impact of the participants, on the one hand, routine is setting in when dealing with the new protection measures, but on the other hand, a certain fatigue about obtaining information over time, especially in winter, has also become noticeable. This is probably due to the overabundance of information and misinformation, so-called “infodemic” [48–50], and is not a singular phenomenon of the participants.

This study showed that more than 90% of the participants indicated that there have been changes in the contact behavior between them and their clients because of the pandemic. The most common reason why services were reduced by providers was related to patient safety. Due to the additional time required to provide care, such as dressing and undressing protective clothing, prioritization of services was necessary to maintain the basic safety of their clients.

The study of Khademi et al. showed that in COVID-19 pandemic the most common reason clients reduced services was fear of becoming infected, which in turn is closely linked to fear of dying, as clients are very aware of their vulnerability [51]. This study showed that, according to the views of outpatient care providers, the feeling of loneliness in connection with the increased social isolation has greatly increased especially in spring and winter. Therefore, in summer, feelings of isolation and loneliness showed significantly lower scores than in spring and winter. This could be since in summer people were more likely to get out of their homes and meet other people outdoors. In addition, the number of people in Switzerland with COVID-19 decreased in summer and the risk of being infected was reduced. So, these physical and psychological effects must be considered, although they have been known for a long time [3, 7–13]. Hence, it can be stated that the COVID-19 pandemic has brought the pandemic of loneliness to surface, which may pose far reaching consequences and major challenges such as mortality risks [11] and increased healthcare expenditure [8, 15–18] to the health care system in the long term, far beyond the COVID-19 pandemic.

Additional goal in this study was to find out if digital technologies were be used and at least counteract loneliness during the pandemic. It turned out that in the view of outpatient care providers the current generation of clients has little or no contact with digital technologies, apart from telephone. From their perspective, the handling of the technologies is either not understood or too complex.

It is also worth considering that 6.8% said that data security is a barrier and may avoid using video calling, messaging and social media, also against the background of actual issues such as fake news [50] and misinformation [49]. In addition, the acquisition and maintenance for the use of these technologies is not affordable for all.

Implications for research and practice can be derived from the results and will be specified.

- Implications for research: The results underpin the phenomena of loneliness and social isolation are common among older people in need of care and their family. These and certain topics as the changes in contact behavior with clients during the pandemic showed that prospective, longitudinal, quantitative data is needed.
  As the results of the use of digital tools show, research on the further development of existing tools (e.g., telephone, video calling) would be desirable. On the one hand, solutions must be found regarding the (cognitive) abilities of the clients concerned. On the other hand, the tools need to ensure communication between caregivers and clients and their monitoring, e.g., to detect the consequences (disorders such as depression, cognitive disorders, deterioration of health) of loneliness and social isolation.
- Implications for practice: To reach the current older generation better and more closely, which is predominantly the goal of outpatient care, through digital technologies during times of limited social contact, but also beyond, it is not the clients who have to change, but the technologies that must be adapted. There is a need to reduce technology complexity, e.g., facilitating the handling of mobile phone using larger buttons. The visualization must be optimized, so that the clients are able to recognize the contrasts, despite decreasing visual performance [52]. The technology may thus contribute to reducing the feeling of loneliness and social isolation in older adults.
  Furthermore, society and politics must deal with the question whether they want to make these technologies available to all socially disadvantaged persons to reduce health care expenditures by reducing social isolation and the feeling of loneliness.

### Limitations of the study

A sufficient response rate of all participants was recorded, which at 30% exceeds most national studies [40], but the targeted response rate (20%) from private outpatient care providers couldn’t be reached. Since this is a cross-sectional study, causal correlations and conclusions may not be drawn.

An additional limitation points out that only the caregiver’s perception of their clients was investigated, the accuracy of this study must be verified by comparing it with the perceptions of clients and families.

The presented study used an instrument that was developed by experts but not validated. The validation of the instrument will be considered in the future. Furthermore, social isolation and loneliness are complex and multidimensional concepts, but were assessed in this study using a single item each. Thus, more expansive measures should examine those constructs in future research.

## Conclusions

Our study describes that clients in Switzerland in the view of outpatient caregivers were and are severely affected by contact limitations due to the COVID-19 pandemic, and to date have been unable to reduce social isolation and loneliness through digital technologies. With the pandemic still ongoing and the impact of social isolation and loneliness beyond the pandemic, these findings point to the urgent need to invest in digital technologies for clients in home care. Accordingly, research should focus on the development of appropriate digital technologies.

## Supplementary Information

**Additional file 1: Table S1.** Results of the analyses.

## Data Availability

Data are available from the corresponding author upon reasonable request and with permission of Ethics Committee Zurich, Zurich, Switzerland.
