# Supplementary material for: Use of digital technologies to combat loneliness and social isolation: a cross-sectional study in Swiss outpatient care during COVID-19 pandemic
Source: BMC Nurs. 2022 Jul 8;21:181. doi: 10.1186/s12912-022-00946-7 (PMC9263432; doi:10.1186/s12912-022-00946-7)
Supplement: Supplementary file 1 — Additional file 1: Table S1. Results of the analyses. [file 12912_2022_946_MOESM1_ESM.docx]

| In the spring of 2020, there was a pandemic plan on what I should do in the event of a pandemic outbreak. | | *n* = 378  1 = 192/ 207.89  2 = 46/ 175.08  3 = 140/ 169.03  *χ*^2^(2) = 11.685, ***p* = .003** z_(3-2)_ = 0.334, *p =* 1.000 z_(3-1)_ = 3.276, ***p* = .003**, r = 0.2  z_(2-1)_ = 1.872, *p* = .184 | | *n* = 365  1 = 59/ 172.70/ 2.28  2 = 306/ 184.99/ 3.05  *U* = 8419.500  z *= -*0.838  *p* = .402 | *n* = 365  1 = 87/ 199.76  2 = 69/ 180.49  3 = 55/ 162.92 | | 4 = 70/ 181.81  5 = 41/ 195.94  6 = 30/ 164.62  7 = 13/ 177.08 | Pandemic plan is availabe in...  1 = spring 2020  2 = winter 2020  *t*(371)_(_*_1-2)_ = -*13.845, *n* = 372, ***p* < .001**, *d* = 0.7 |
| --- | --- | --- | --- | --- | --- | --- | --- | --- |
|  |  |  |  |  | *χ*^2^(6) = 6.086, *p* = .414 | | |  |
| Now in the winter of 2020, there is a pandemic plan for what I should do if the number of COVID-19 infected people increases. | | *n* = 375  1 = 189/ 207.24  2 = 45/ 193.53  3 = 141/ 160.44  *χ*^2^(2) = 17.209, ***p* < .001** z_(3-2)_ = 1.898, *p =* .173 z_(3-1)_ = 4.130, ***p* < .001**, r = 0.2  z_(2-1)_ = 1.872, *p* = .417 | | *n* = 362  1 = 58/ 172.83/ 3.97  2 = 304/ 183.15/ 4.01  *U* = 8313.000  z *= -*0.733  *p* = .464 | *n* = 362  1 = 87/ 174.51  2 = 69/ 178.95  3 = 55/ 169.26 | | 4 = 68/ 179.05  5 = 40/ 232.25  6 = 30/ 176.25  7 = 13/ 162.35 |  |
|  |  |  |  |  | *χ*^2^(6) = 12.607, ***p* = .050** | |  |  |
|  |  |  |  |  | z_(7-3)_ = 0.228, *p* = 1.000  z_(7-1)_ = 0.416, *p* = 1.000  z_(7-6)_ = 0.426, *p* = 1.000  z_(7-2)_ = 0.558, *p* = 1.000  z_(7-4)_ = 0.561, *p* = 1.000  z_(7-5)_ = 2.226, *p* = .546  z_(3-1)_ = 0.310, *p* = 1.000  z_(3-6)_ = -0.313, *p* = 1.000  z_(3-2)_ = 0.545, *p* = 1.000  z_(3-4)_ = -0.549, *p* = 1.000 z_(3-5)_ = -3.082, ***p* = .043**, r = 0.3 | z_(1-6)_ = -0.083, *p* = 1.000  z_(1-2)_ = -0.280, *p* = 1.000  z_(1-4)_ = -0.285, *p* = 1.000  z_(1-5)_ = -3.073, ***p* = .045**, r = 0.3  z_(6-2)_ = 0.125, *p* = 1.000  z_(6-4)_ = 0.125, *p* = 1.000  z_(6-5)_ = 2.357, *p* = .387  z_(2-4)_ = -0.006, *p* = 1.000  z_(2-6)_ = -2.727, *p* = .134  z_(4-5)_ = -2.714, *p* = .139 | |  |
| In the spring 2020, at the beginning of the pandemic, sufficient protective clothing (e.g., mouth guards, goggles) was available. | | *n* = 381  1 = 192/ 203.83  2 = 46/ 186.58  3 = 143/ 175.20  *χ*^2^(2) = 6.786, ***p* = .034** z_(3-2)_ = 0.670, *p =* 1.00 z_(3-1)_ = 2.585, ***p* = .029**, r = 0.1  z_(2-1)_ = 1.872, *p* = .417 | | *n* = 367  1 = 59/ 187.42/ 1.85  2 = 308/ 183.34/ 1.87  *U* = 8884.000  z *= -*0.297  *p* = .766 | *n* = 367  1 = 87/ 183.67  2 = 69/ 165.62  3 = 56/ 163.96 | | 4 = 71/ 196.31  5 = 41/ 202.89  6 = 30/ 172.70  7 = 13/ 269.38 | Protective clothing is available in...  1 = spring 2020  2 = summer 2020  3 = winter 2020  *t*(379)_(_*_1-2)_ =* -30.681, *n* = 380, ***p* < .001**, *d* > 0.8  *t*(378)_(_*_1-3)_ =* -35.229, *n* = 379, ***p* < .001**, *d* > 0.8  *t*(377)_(_*_2-3)_ =* -9.496, *n* = 378, ***p* < .001**, *d* = 0.5 |
|  |  |  |  |  | *χ*^2^(6) = 18.216, ***p* = .006** | |  |  |
|  |  |  |  |  | z_(3-2)_ = 0.096, *p* = 1.000  z_(3-6)_ = -0.400, *p* = 1.000  z_(3-1)_ = 1.192, *p* = 1.000  z_(3-4)_ = -1.875, *p* = 1.000  z_(3-5)_ = -1.962, *p* = 1.000  z_(3-7)_ = -3.547, ***p* = .008**, r = 0.4  z_(2-6)_ = -0.335, *p* = 1.000  z_(2-1)_ = 1.160, *p* = 1.000  z_(2-4)_ = -1.880, *p* = 1.000  z_(2-4)_ = -0.549, *p* = 1.000  z_(2-5)_ = -1.958, *p* = 1.000 | z_(2-7)_ = -3.554, ***p* = 0.008**, r = 0.4  z_(6-1)_ = 0.537, *p* = 1.000  z_(6-4)_ = 1.123, *p* = 1.000  z_(6-5)_ = 1.301, *p* = 1.000  z_(6-7)_ = -3.016, *p* = .054  z_(1-4)_ = -0.818, *p* = 1.000  z_(1-5)_ = -1.051, *p* = 1.000  z_(1-7)_ = -2.985, *p* = 0.059  z_(4-5)_ = -0.347, *p* = 1.000  z_(4-7)_ = -2.509, *p* = .254  z_(5-7)_ = -2.164, *p* = .640 | |  |
| With time, over the summer 2020 was enough protective clothing. | | *n* = 380  1 = 191/ 199.47  2 = 46/ 181.41  3 = 143/ 181.45  *χ*^2^(2) = 2.918, *p* = .233 | | *n* = 366  1 = 59/ 156.44/ 3.61  2 = 307/ 188.70/ 3.93  *U* = 7460.000  z *= -*2.292  ***p* = .022** | *n* = 366  1 = 87/ 167.58  2 = 69/ 181.39  3 = 56/ 175.79 | | 4 = 71/ 180.59  5 = 41/ 228.44  6 = 29/ 186.02  7 = 13/ 202.96 |  |
|  |  |  |  |  | *χ*^2^(6) = 11.662, *p* = .070 | | |  |
| Now in the winter of 2020, there is enough protective clothing. | | *n* = 380  1 = 191/ 196.58  2 = 46/ 165.74  3 = 143/ 190.34  *χ*^2^(2) = 3.772, *p* = .152 | | *n* = 366  1 = 59/ 180.52/ 4.39  2 = 307/ 184.07/ 4.34  *U* = 8880.500  z *= -*0.269  *p* = .788 | *n* = 366  1 = 87/ 187.33  2 = 69/ 181.51  3 = 56/ 178.67 | | 4 = 71/ 162.23  5 = 41/ 208.90  6 = 29/ 189.62  7 = 13/ 211.65 |  |
|  |  |  |  |  | *χ*^2^(6) = 8.378, *p* = .212 | | |  |
| In the spring of 2020, I was afraid of getting infected. | | *n* = 379  1 = 193/ 193.80  2 = 46/ 187.08  3 = 140/ 185.72  *χ*^2^(2) = 0.501, *p* = .778 | | *n* = 368  1 = 60/ 170.11/ 2.73  2 = 308/ 187.30/ 2.99  *U* = 8880.500  z *= -*0.269  *p* = .788 | *n* = 368  1 = 89/ 213.87  2 = 69/ 177.88  3 = 55/ 180.37 | | 4 = 71/ 166.40  5 = 41/ 196.46  6 = 30/ 147.20  7 = 13/ 183.23 | Afraid of getting infected in...  1 = spring 2020  2 = winter 2020  *t*(375)_(_*_1-2)_ =* 5.085, *n* = 376, ***p* < .001**, *d* = 0.3 |
|  |  |  |  |  | *χ*^2^(6) = 14.020, ***p* = .029** | | |  |
|  |  |  |  |  | z_(6-4)_ = 0.848, *p* = 1.000  z_(6-2)_ = 1.349, *p* = 1.000  z_(6-3)_ = 1.405, *p* = 1.000  z_(6-7)_ = -1.043, *p* = 1.000  z_(6-5)_ = 1.972, *p* = 1.000  z_(6-1)_ = 3.037, ***p* = .050**, r = 0.3  z_(4-2)_ = 0.653, *p* = 1.000  z_(4-3)_ = 0.748, *p* = 1.000  z_(4-7)_ = 0.536, *p* = 1.000  z_(4-6)_ = -1.474, *p* = 1.000  z_(4-1)_ = 2.869, *p* = .087 | z_(2-3)_ = -0.133, *p* = 1.000  z_(2-7)_ = -0.170, *p* = 1.000  z_(2-5)_ = -0.906, *p* = 1.000  z_(2-1)_ = 2.159, *p* = .650  z_(3-7)_ = -0.089, *p* = 1.000  z_(3-5)_ = -0.750, *p* = 1.000  z_(3-1)_ = 1.878, *p* = 1.000  z_(7-5)_ = 0.400, *p* = 1.000  z_(7-1)_ = 0.992, *p* = 1.000  z_(7-1)_ = 0.992, *p* = 1.000  z_(5-1)_ = 0.887, *p* = 1.000 | |  |
| Now in the winter of 2020, I'm afraid of getting infected. | | *n* = 377  1 = 192/ 186.05  2 = 46/ 187.68  3 = 139/ 193.51  *χ*^2^(2) = 0.407, *p* = .816 | | *n* = 366  1 = 60/ 177.53/ 2.55  2 = 306/ 184.67/ 2.64  *U* = 8820.000  z *= -*0.490  *p* = .624 | *n* = 366  1 = 88/ 200.82  2 = 69/ 183.17  3 = 56/ 177.87 | | 4 = 70/ 178.33  5 = 41/ 190.62  6 = 29/ 147.24  7 = 13/ 178.58 |  |
|  |  |  |  |  | *χ*^2^(6) = 6.643, *p* = .355 | | |  |
| By spring 2020, I felt confident in using hygiene protection concepts to prevent infection or transmission of the virus. | | *n* = 380  1 = 194/ 197.86  2 = 46/ 202.47  3 = 140/ 176.38  *χ*^2^(2) = 3.941, *p* = .139 | | *n* = 369  1 = 60/ 192.21/ 3.27  2 = 309/ 183.60/ 3.15  *U* = 8837.500  z *= -*0.588  *p* = .557 | *n* = 369  1 = 89/ 157.19  2 = 69/ 186.35  3 = 56/ 207.99 | | 4 = 71/ 181.96  5 = 41/ 208.04  6 = 30/ 194.80  7 = 13/ 190.54 | Feeling confident using hygiene protection concepts in...  1 = spring 2020  2 = winter 2020  *t*(377)_(_*_1-2)_ =* -9.902, *n* = 378, ***p* < .001**, *d* = 0.5 |
|  |  |  |  |  | *χ*^2^(6) = 11.525, *p* = .073 | | |  |
| Now in the winter of 2020, I feel confident in using hygiene protection concepts to prevent infection or transmission of the virus. | | *n* = 378  1 = 194/ 198.32  2 = 45/ 211.32  3 = 139/ 170.12  *χ*^2^(2) = 8.199, ***p* = .017** z_(3-1)_ = 2.440, *p =* .044, r = 0.1 z_(3-2)_ = 2.309, *p* = .063  z_(1-2)_ = -0.755, *p* = 1.000 | | *n* = 367  1 = 60/ 178.08/ 3.78  2 = 307/ 185.16/ 3.82  *U* = 8855.000  z *= -*0.496  *p* = .620 | *n* = 367  1 = 89/ 159.52  2 = 69/ 179.44  3 = 55/ 200.47 | | 4 = 71/ 181.25  5 = 40/ 213.80  6 = 30/ 202.40  7 = 13/ 186.92 |  |
|  |  |  |  |  | *χ*^2^(6) = 11.369, *p* = .078 | | |  |
| In the spring of 2020, I kept my knowledge of the virus up to date. | | *n* = 379  1 = 194/ 190.27  2 = 46/ 192.16  3 = 139/ 188.90  *χ*^2^(2) = 0.041, *p* = .979 | | *n* = 368  1 = 60/ 172.13/ 4.27  2 = 308/ 186.91/ 4.35  *U* = 8498.000  z *= -*1.100  *p* = .271 | *n* = 368  1 = 89/ 169.62  2 = 69/ 194.46  3 = 55/ 205.07 | | 4 = 71/ 162.76  5 = 41/ 206.21  6 = 30/ 188.17  7 = 13/ 188.23 | Keeping knowledge up to date in...  1 = spring 2020  2 = winter 2020  *t*(375)_(_*_1-2)_ =* 6.545, *n* = 376, ***p* < .001**, *d* = 0.3 |
|  |  |  |  |  | *χ*^2^(6) = 11.396, *p* = .077 | | |  |
| Now in the winter of 2020, I always keep my knowledge about the virus up to date. | | *n* = 377  1 = 192/ 188.90  2 = 46/ 191.75  3 = 139/ 188.23  *χ*^2^(2) = 0.042, *p* = .979 | *n* = 366  1 = 60/ 173.65/ 3.93  2 = 306/ 185.43/ 4.10  *U* = 8589.000  z *= -*0.846  *p* = .398 | | *n* = 366  1 = 88/ 174.93  2 = 69/ 189.20  3 = 56/ 192.62 | | 4 = 70/ 172.44  5 = 41/ 188.17  6 = 30/ 198.30  7 = 12/ 182.58 |  |
|  |  |  |  |  | *χ*^2^(6) = 3.019, *p* = .806 | | |  |
| Clients felt lonely in spring 2020 | - | | - | | - | | | Loneliness of the clients in...  1 = spring 2020  2 = summer 2020  3 = winter 2020  *t*(373)_(_*_1-2)_ =* 15.214, *n* = 374, ***p* < .001**, *d* = 0.8  *t*(369)_(_*_1-3)_ = -*1.196, *n* = 370, *p* = .232  *t*(368)_(_*_2-3)_ =* -16.507, *n* = 369, ***p* < .001**, *d* < 0.8 |
| Clients felt lonely in summer 2020 | - | | - | | - | | |  |
| Clients felt lonely in winter 2020 | - | | | - | - | | |  |
| Clients were isolated in spring 2020 | - | | | - | - | | | Social isolation of the clients in...  1 = spring 2020  2 = summer 2020  3 = winter 2020  *t*(372)_(_*_1-2)_ =* 15.736, *n* = 373, ***p* < .001**, *d* = 0.8  *t*(372)_(_*_1-3)_ =* 0.569, *n* = 373, *p* = .570  *t*(373)_(_*_2-3)_ =* -16.652, *n* = 369, ***p* < .001**, *d <* 0.8 |
| Clients were isolated in summer 2020 | - | | | - | - | | |  |
| Clients were isolated in winter 2020 | - | | | - | - | | |  |
| **Legend:**  ^1^ Since the gender "diverse" was only given by one participant, it was interpreted as "missing value" for the group comparison. | | | | | | | | |
